# Hyperoxia accelerates Fas-mediated signaling and apoptosis in the lungs of *Legionella pneumophila *pneumonia

**DOI:** 10.1186/1756-0500-4-107

**Published:** 2011-04-06

**Authors:** Tsuneharu Maeda, Soichiro Kimura, Tetsuya Matsumoto, Yoshinari Tanabe, Fumitake Gejyo, Keizo Yamaguchi

**Affiliations:** 1 Department of Microbiology and Infectious Diseases, Toho University Faculty of Medicine, Tokyo 143-8540, Japan; 2 Division of Respiratory Medicine and Infection Control and Prevention Niigata University Graduate School of Medical and Dental Sciences, Niigata University Medical School, Niigata 951-8510, Japan; 3 Department of Microbiology, Tokyo Medical University, Tokyo, Japan

## Abstract

**Background:**

Oxygen supplementation is commonly given to the patients with severe pneumonia including *Legionella *disease. Recent data suggested that apoptosis may play an important role, not only in the pathogenesis of *Legionella *pneumonia, but also in oxygen-induced tissue damage. In the present study, the lethal sensitivity to *Legionella *pneumonia were compared in the setting of hyperoxia between wild-type and Fas-deficient mice.

**Findings:**

C57BL/6 mice and B6.MRL-*Fas^lpr ^*mice characterized with Fas-deficiency were used in this study. After intratracheal administration of *L. pneumophila*, mice were kept in hyperoxic conditions (85-90% O_2 _conc.) in an airtight chamber for 3 days. Bone-marrow derived macrophages infected with *L. pneumophila *were also kept in hyperoxic conditions. Caspase activity and cytokine production were determined by using commercially available kits. Smaller increases of several apoptosis markers, such as caspase-3 and -8, were demonstrated in Fas-deficient mice, even though the bacterial burdens in Fas-deficient and wild type mice were similar. Bone-marrow derived macrophages from Fas-deficient mice were shown to be more resistant to *Legionella*-induced cytotoxicity than those from wild-type mice under hyperoxia.

**Conclusions:**

These results demonstrated that Fas-mediated signaling and apoptosis may be a crucial factor in the pathogenesis of *Legionella *pneumonia in the setting of hyperoxia.

## Introduction

*Legionella pneumophila *is a Gram-negative intracellular pathogen, and often causes a severe and life-threatening pneumonia [1,2]. *Legionella *pneumonia is frequently complicated with acute lung injury and acute respiratory distress syndrome, which exaggerates the severity of this disease [3,4]. Despite aggressive supportive care, including antibiotic therapy and oxygen supplementation, high mortality rates have been reported, especially in immunocompromised patients [3,5,6].

Multiplication of *L. pneumophila *in lung tissue is shown in several types of host cells, including macrophages, monocytes, and alveolar epithelial cells [7-9]. Previous reports showed that *L. pneumophila *can induce apoptosis in macrophages and alveolar epithelial cells [10-12]. Apoptosis is a highly regulated process of cell death that is required for development and homeostasis of multicellular organisms in physiological condition [13,14]. The two apoptosis pathways are the extrinsic and intrinsic pathways [15]. The extrinsic pathway is initiated by stimulation of the transmembrane death receptors by specific ligands such as Fas, and then procaspase 8 is proteolytically activated. Procaspase 8 proteolytically activates effectors proteins such as caspase 3 and caspase 7. The intrinsic pathway is usually activated by apoptotic signals such as cell stress. The stimuli cause changes to the inner mitochondrial permeability and then cytochrome c is released into the cytosol. The release of cytochrome c stimulates apoptosome formation, and can lead to the activation of caspase-9, and then of caspase-3. A wide variety of pathogens were reported to modulate the host cell-death pathway by direct interaction with key components of the apoptosis machinery of the host [16,17]. Unfortunately, how apoptosis is involved in the pathogenesis of *Legionella *disease is incompletely understood.

Supplementation with oxygen is a critical part of supportive care for severe pneumonia, including *Legionella *infection, as an adjunct to intensive antibiotic chemotherapy. Unfortunately, prolonged administration of oxygen itself may be associated with exaggeration of lung damage in these patients [18]. Recent progress in this field suggests an important role for apoptosis in hyperoxia-mediated lung injury in a variety of cells, such as epithelial cells and microvascular endothelial cells [19-21]. In a previous study, we found that hyperoxia caused the development of acute lung injury and lethality in mice with *Legionella *pneumonia [22]. However, only limited information is available for about the significance of hyperoxia in pathogenesis of *Legionella *pneumonia, especially from the standpoint of apoptosis in the infected lungs.

In the present study, we examined *Legionella *pneumonia associated with Fas-mediated apoptosis in the setting of hyperoxia. To understand the mechanism of lethal sensitivity, several apoptosis markers including caspase-3, -8, and cytokine productions were explored.

## Materials and methods

### Animals

Specific pathogen-free 6-to 10-wk-old C57BL/6 mice (Charles River Japan, Tokyo, Japan) and B6.MRL-*Fas^lpr ^*mice characterized with Fas-deficiency (The Jackson Laboratory, Bar Harbor, ME) were used. Fas-deficient mice were bred in our animal facility. All mice were housed in specific pathogen-free conditions within the animal care facility at Laboratory Animal Research Center of Toho University of Medicine until the day of sacrifice (approved number #169).

### Culture of *Legionella *organism and inoculation of bacteria in vivo

We used a clinical isolate of *L. pneumophila *Suzuki strain (serogroup 1) for all experiments. *N*-(2-acetamido)-2-aminoethanesulfonic acid (Sigma-Aldrich, St. Louis, MO)-buffered yeast extract (BYE) broth supplemented with L-cysteine (0.4 μg/ml) and ferric nitrate (0.135 μg/ml) was used as liquid medium (BYE-broth). To prepare solid medium, activated charcoal (2 μg/ml) and agar (15 μg/ml) were added to liquid medium (BCYE agar). *L. pneumophila *was incubated on BCYE-agar for 4 days at 37°C. A single colony was transferred to 3 ml of BYE-broth, and was then incubated overnight at 37°C with constant shaking. The bacterial suspension was again transferred to fresh BYE-broth, and incubated overnight under the same conditions. The bacterial suspension was diluted to the desired concentrations in saline according to a standard of absorbencies based on known CFU. Animals were anesthetized intraperitoneally with 2.2 and 11.1 mg/kg xylazine and ketamine, respectively. The trachea was exposed and 30 μl of inoculum was administrated via a sterile 26 gauge needle. The skin incision was closed with surgical staples.

### Oxygen exposure in vivo

After intratracheal (i.t.) administration of bacteria, one group of mice was kept in hyperoxic conditions in an airtight chamber for 3 days, whereas another group was placed in room air conditions according to our previous studies [22,23]. For hyperoxic exposure, the oxygen concentration in the chamber was kept between 85 and 90% by a constant flow gas, which was monitored with an in-line oxygen analyzer (O_2 _CONTROLLER model MC-70, IIJIMA Electronics Corp. Japan) [22]. Carbon dioxide levels in the chamber were maintained at 0.03-0.04% during the course of experiments. Both groups of mice were fed food and water ad lib and kept on a 12-h dark night cycle at room temperature.

### Lung harvesting for analysis

At designated time points, mice were sacrificed using CO_2 _asphyxia. Before the lung removal, the pulmonary vasculature was perfused with 1 ml of saline, via the right ventricle. Whole lungs were then harvested for assessment of bacterial number, cytokine protein expression, and apoptosis [22]. After the removal of whole lungs, they were homogenized in 1.0 ml of saline using a tissue homogenizer (Omni TH HOMOGENIZER, YAMATO scientific products Co,. Ltd., Tokyo) under a vented hood. Portions of homogenates (10 μl) were inoculated on agar after serial 1/10 dilutions with saline. The remaining homogenates were centrifuged at 2000 rpm for 10 min in 4°C. Supernatants were collected, and passed through a 0.45-μm filter (KANTO CHEMICAL CO,. INC, Tokyo). These samples were stored at -20°C for further analysis.

### Oxygen exposure in vitro

The BM-derived macrophages infected with *Legionella *in 96-well plates were kept in hyperoxic conditions in a multi gas incubator (ASTEC Co,. Ltd, TOKYO) and incubated at 37°C in 85-90% of oxygen and 5% of CO_2_. In normoxia experiments, the plate was kept in 5% CO_2 _incubator during the course of experiments.

### Preparation of bone-marrow derived macrophage

Murine bone-marrow (BM) derived macrophages were prepared from control C57BL/6 and Fas-deficient mice by the following method [24]. BM cells were extracted from femurs, and suspended in RPMI with L-glutamine added 10% fetal bovine serum (10 ml per mouse). The BM cells in 2 ml were plated in the cell incubate dishes (BIOCOAT) and then added 8 ml (per dish) of RPMI with L-glutamine containing L929 cell conditioned medium (20%), fetal bovine serum (10%), penicillin (100 U/ml) and streptomycin (100 μg/ml), and 0.04% of M-CSF. The dishes were incubated at 37°C in humidified atmosphere of 5% CO_2_. After 4 days, BM-derived macrophages in primary culture were harvested and identified morphologically by the adhesion of bottom of dish. The supernatant medium was removed from the dishes and replaced with 5 ml fresh medium (RPMI with L-glutamine added 10% fetal bovine serum), and adherent macrophages peeled with a rubber brush. Then, the cells in the medium was collected and seeded at a concentration of 10^5 ^cell/well in a 96-well plate. After overnight incubation, each well was washed with the above medium and infected with the desired concentrations of bacteria. After 2 hours of incubation, the wells were washed with the medium to remove un-attached bacteria [25], and then the cells were incubated in 5% CO_2 _with or without hyperoxia. After macrophages were infected with *L. pneumophila*, shrunken cells (apoptotic cells) but not necrosis cells were observed by microscopic analysis (data not shown).

### Determination of caspases activity

To evaluate induction of apoptosis, caspase-3 and -8 activities were determined in the homogenates of the lungs of wild-type and Fas-deficient mice with pneumonia. To confirm the activities of caspase cascade in apoptosis, Caspase-3 and -8 activities were determined by a colorimetric assay (R&D Systems, Minneapolis, MN). The data were expressed as a fold increase, comparing to those of control mice (*n *= 5).

### Murine cytokines ELISA

Murine cytokines (TNF-α, IL-6, IL-12) were quantitated using specific ELISAs (R&D systems, Minneapolis, MN) according to manufacturerers' instructions.

### Cell viability by Tetracolor®

Incubated bone marrow-derived macrophages (after Trypsin-EDTA treatment) were added in 96-well plates (1 × 10^5 ^/well), and incubated for 24-48 hr at 37°C. 10 μl of Tetracolor ONE^® ^were added in each well and incubated for 24 hr at 37°C. Measurement was determined by ELISA.

### Statistical analysis

Statistical significance was determined using the unpaired, two-tailed alternate Welch *t *test. Calculations were performed using JSTAT for Windows (Masato Sato, Japan). Statistic analyses of survival curves were performed by using Delta Graph (SPSS Inc.).

## Results

### Bacterial number in the lungs of mice with *Legionella *pneumonia

**Figure 1:**
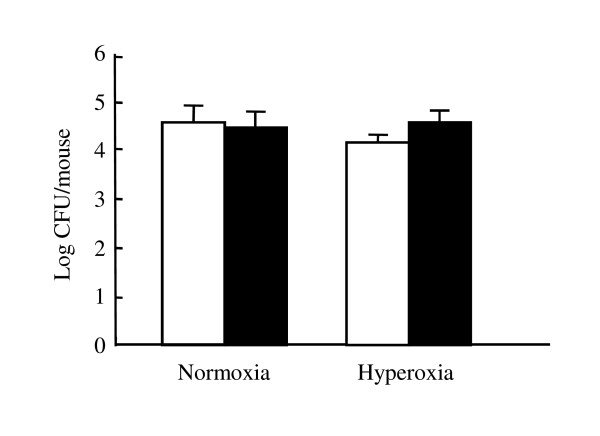
**Bacterial numbers in the lungs of mice with *L. pneumophila***. Wild-type and Fas-deficient mice were infected intratracheally with approximately 10^6 ^CFU/mouse of *L. pneumophila*. The mice of hyperoxia group were kept in hyperoxic condition. The lungs were removed and homogenized at 48 hours after the infection. The lung homogenates were incubated on BCYE-α agar after serial 10-fold dilution for 4 days. The white column represents the bacterial number in the lungs of wild-type mice whereas the filled column represents the results of Fas-deficient mice (n = 5).

It has been reported that Fas-deficient mice may be more resistant to *Legionella *pneumonia infection in the setting of hyperoxia [22]. To explore the mechanisms of lower lethality of Fas-deficient mice with *Legionella *pneumonia in hyperoxia, we examined bacterial numbers in the lungs of wild-type and Fas-deficient mice. The results demonstrated clearly no differences of the bacterial numbers in the lungs between wild-type and Fas-deficient mice, in both settings of normoxia and hyperoxia (Figure 1). In in-vitro experiments using BM-derived macrophages, we observed no differences of bacterial numbers in macrophages derived from wild-type and Fas-deficient mice, in despite of the condition normoxia and hyperoxia (data not shown). These results suggested that bacterial number may not be a critical factor responsible for a mechanism of higher resistance of Fas-deficient mice against *Legionella *pneumonia in the setting of hyperoxia.

### Effects of hyperoxia on Legionella-induced apoptosis in the lungs of mice

**Figure 2:**
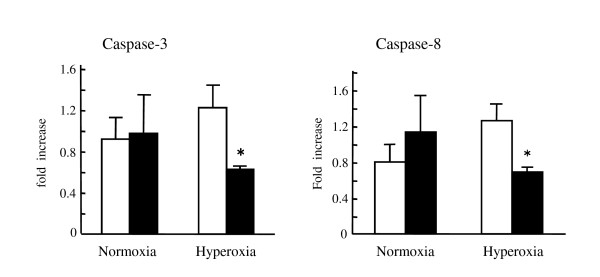
**Caspase-3 and caspase-8 in the lungs of mice with *L. pneumophila***. Wild-type and Fas-deficient mice were infected intratracheally with approximately 10^6 ^CFU/mouse of *L. pneumophila*. One group was kept in room air condition and another group was placed in a hyperoxic condition. Lungs were removed and homogenized at 48 hours after the infection. Caspase activity was quantified by the ELISA, as described in materials and methods. The white column represents the results of the lungs of wild-type mice whereas the filled column represents the results of Fas-deficient mice (n = 5). *P<0.01, compared to those of wild-type mice.

In a previous study, we found that significantly lower values of histone-associated DNA fragments were observed in the lungs of Fas-deficient mice in hyperoxia compared to in normoxic condition [22]. Therefore, we examined the roles of apoptosis of *Legionella *pneumonia in wild-type and Fas-deficient mice. We explored the activities of caspase-3 (an end-stage marker for apoptosis) and caspase-8 (a marker of apoptosis associated with the Fas-signaling system) in the lungs of mice. For both markers, there were no differences in the lungs between wild-type and Fas-deficient mice when they were kept in normoxic condition (Figure 2). On the other hand, significantly lower values of caspase-3 and caspase-8 were observed in Fas-deficient mice under hyperoxic condition. These results suggested less induction of apoptosis in the lungs of Fas-deficient mice kept in hyperoxia, as evidenced by several factors, such as histone-associated DNA fragments and caspase-3, caspase-8.

### Inflammatory cytokines in the lungs of wild-type and Fas-deficient mice

**Figure 3:**
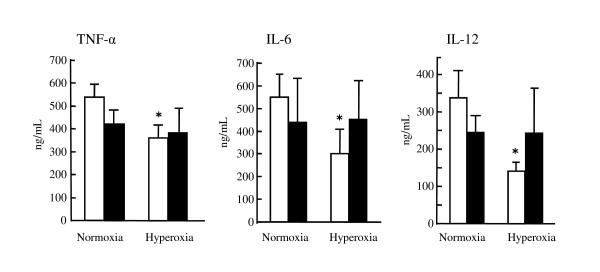
**Cytokines in the lungs of mice with *L. pneumophila***. Wild-type (n = 5) and Fas-deficient mice (n = 5) were infected intratracheally with approximately 10^6 ^CFU/mouse of *L. pneumophila*. One group was kept in room air condition and another group was placed in hyperoxic condition. Lungs were removed and homogenized at 48 hours after the infection. TNF-α, IL-6 and IL-12 were quantified by the ELISA, as described in materials and methods. The white column represents the results of the lungs of wild-type mice whereas the filled column represents the results of Fas-deficient mice (n = 5). *P<0.05, compared to wild-type mice in room air condition.

We examined inflammatory cytokines, such as TNF-a, IL-6 and IL-12, in the lungs of wild-type and Fas-deficient mice kept in normoxic or hyperoxic condition (Figure 3). In control mice, there was a trend of lower values of these factors in the setting of hyperoxia. When the values were compared in wild-type and Fas-deficient mice, statistically significant differences were not demonstrated between wild-type and Fas-deficient mice in both normoxic and hyperoxic conditions.

### Viability of BM-derived macrophages infected with *L. pneumophila*

**Figure 4:**
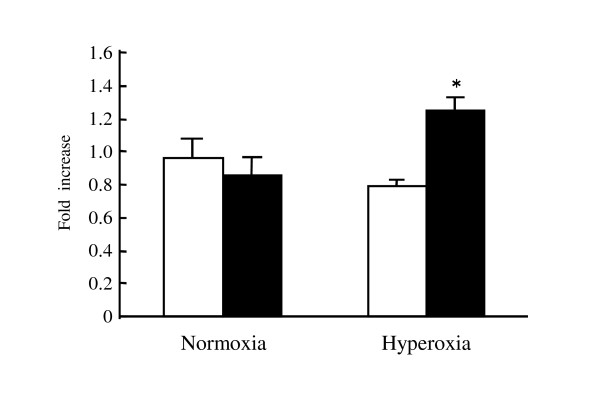
**BM-derived macrophages viability after infection with *L. pneumophila***. BM-derived macrophages from wild-type and Fas-deficient mice were prepared as described in materials and methods. The cells were infected with *L. pneumophila *at MOI of 1 and then kept in 5% CO_2 _or 5%CO_2 _plus hyperoxia. The viability of cells was examined 24 hours after the infection. The white column is the results of the wild-type mice whereas the filled column indicated results of Fas-deficient mice (n = 5).

Finally, viability of BM-derived macrophages from wild-type and Fas-deficient mice was compared after the infection with *L. pneumophila *(Figure 4). In normoxic condition, there were no differences of viability of macrophages between wild-type and Fas-deficient mice. On the other hand, significantly higher viability was demonstrated in BM-derived macrophages from Fas-deficient mice, when the infected cells were kept in hyperoxia. Shrunken cells were frequently observed in wild type mice in hyperoxia by microscopic analysis (data not shown). These data suggested that BM-derived macrophages from Fas-deficient mice may be more resistant to *L. pneumophila*-induced cytotoxicity in the setting of hyperoxia.

## Discussion

Apoptosis can be triggered through two major pathways, one implicating death receptor ligation (e.g. Fas and TNF-α) and the other is the release of cytochrome c from mitochondria [15]. In the lungs and pulmonary cells, several investigators have reported that the Fas-mediated signaling system may be an important developmental regulator for induction of apoptosis [26,27]. These "death-inducing" receptors recruit the death-inducing signaling complex through interaction with adaptor proteins, such as Fas associated death domain protein (FADD). In turn, FADD recruits caspase-8. Although a variety of known and unknown anti-apoptotic and pro-apoptotic factors may play a role, subsequent activation of proteases through mitochondria-dependent or -independent pathways results in activation of the key effector enzyme, caspase-3. Activation of executioner caspases results in DNA laddering, characteristic of apoptotic cells, and to the cleavage of cytoskeletal proteins. We observed less exaggerated changes of the initiator caspase-8 and effector caspase -3 and DNA fragmentation (histone-associated DNA fragments) in the lungs of Fas-deficient mice under hyperoxia. However, how hyperoxia and *Legionella *infection are interacting and exaggerating each other in the lungs of mice, particularly the molecular role of Fas remains unknown. The future use of animal models deficient in another protein of the apoptotic pathway may lead to a clearer understanding of this.

Apoptosis has been described as a major death mechanism in hyperoxia-induced lung injury in various animal models in vivo [18,28-31]. Barazzone and collaborators have reported increased pulmonary Fas mRNA levels in adult mice exposed to 100% oxygen, although they failed to demonstrate survival advantage in Fas-deficient *lpr *mice exposed to hyperoxia [21]. In concordance with this finding, De Paepe and associates have demonstrated that Fas gene silencing by siRNAs significantly reduced hyperoxia-induced apoptosis [32]. In contrast, several investigators have reported that other apoptotic signaling pathways, such as mitochondrial pathways and pro-apoptotic factor Bax, may participate in the regulation of hyperoxia-induced cell death [33-35]. Taken together, these data suggest that Fas-mediated signaling and apoptosis, in addition to other intrinsic apoptosis-regulating mechanisms, may be involved in the pathogenesis of hyperoxia-induced lung injuries.

* L. pneumophila *has been shown to induce apoptosis within macrophages, monocytic cells and alveolar epithelial cells [10,12]. Although the mechanisms of *L. pneumophila*-associated apoptosis are not well understood, there are at least two possibilities. The first is that *L. pneumophila *may induce apoptosis through ligation of death receptors, such as Fas and TNF-receptor, by bacterial surface components or by secreted bacterial factors. Another possibility is that translocation of apoptotic factor(s), probably through the Dot/Icm secretion machinery of bacteria, followed by direct activation of caspases within the cytosol. Previously, Gao and Abu-Kwaik demonstrated that activation of caspase-3, but not caspase-1, is essential for apoptosis induced upon infection by *L. pneumophila *[11], but the initial events of caspase induction remained unclear at that time. Molmeret and associates have shown that caspase-3 activation by *L. pneumophila *is independent of all the known apoptotic pathways, such as the extrinsic and intrinsic, that converge on the activation of caspase-3 [36]. Neumeister and collaborators have shown that *L. pneumophila *induced comparable apoptosis in Fas-positive and Fas-negative human monocytic cells, in which correlated caspase activation and mitochondrial cytochrome *c *release were demonstrated [37]. It seems likely that apoptosis induced by *L. pneumophila *is independent of the interaction between bacteria or bacterial products with the death receptor Fas. Furthermore, Fischer and associates have reported that caspase-8-deficient Jurkat cells still underwent cell death, suggesting that caspase-8 is not the relevant initiator caspase in *L. pneumophila-*induced apoptosis [38]. These data suggested that it may be important to consider the possibility of activation of apoptosis-related enzymes in a retrograde fashion in *L. pneumophila-*induced apoptosis.

Several cytokines, chemokines and growth factors have been reported to play a critical role in the pathogenesis of hyperoxia-related lung injury. These include IL-6, IL-8, IL-11, granulocyte-macrophage colony-stimulating factor (GM-CSF) and keratinocyte growth factor (KGF). In the present study, only limited cytokines were examined in *L. pneumophila-*infected lungs of wild-type and Fas-deficient mice under hyperoxia. The data of TNF-a, IL-6 and IL-12 demonstrated no characteristic changes associated with lethality of mice and alterations in apoptosis markers. Further studies examining other factors, such as IL-11, GM-CSF and KGF, may be important to correctly characterize the roles and significance of cytokines and growth factors in *Legionella *pneumonia under hyperoxia.

The present data demonstrate that BM-macrophages from Fas-deficient mice were more resistant to *L. pneumophila *infection than those from wild-type mice in the setting of hyperoxia. These data are consistent with the survival data and the apoptosis markers in Fas-deficient mice with *L. pneumophila *pneumonia. The Dot/Icm secretion system of *L. pneumophila *may be a crucial factor determining cytotoxic effects of this organism, because bacterial mutants deficient in this system demonstrated a striking reduction of the capacity to cause cytotoxicity through induction of apoptosis [36,39]. Although the mechanisms of this phenomenon remain incompletely known, BM-derived Mφ experiments may provide an opportunity to examine an interaction between Dot/Icm secretion system of *L. pneumophila *and hyperoxia-mediated alterations in host cellular defense systems.

The present data suggest that the blocking of apoptosis, especially the Fas-mediated cascade, may be a potential candidate for a new therapeutic approach against *L. pneumophila *pneumonia under hyperoxia. Although it may be critical to understand the molecular and cellular pathogenesis of the events occurred in the lungs of these individuals, silencing of Fas by siRNA and blocking of Dot/Icm secretion system of *L. pneumophila *may be attractive strategies. IAP (Inhibitor of apoptosis) family proteins such as survivin may be potential candidates for therapy [40]. In addition, antioxidants such as glutathione may be also a potential therapeutic candidate [23,41]. Since *L. pneumophila *pneumonia is still a potentially lethal infectious diseases and significant numbers of the cases are treated with supplementation of high concentrations of oxygen, further investigations are warranted for better understanding of the mechanisms of the diseases and to search for life-saving therapeutic strategies.

## Competing interests

The authors declare that they have no competing interests.

## Authors' contributions

TM was involved in the study concept, data collection, data analysis and prepared the first draft of manuscript. SK participated in conception of study design and revision of manuscript. TM, YT and FG contributed to the revision of the manuscript. YK managed the study and edited the manuscript. All authors have read and approve the final manuscript.
